# Optimizing Door-to-Balloon Time for Patients Undergoing Primary Percutaneous Coronary Intervention at King Abdullah Medical City

**DOI:** 10.1155/2024/9823144

**Published:** 2024-08-31

**Authors:** Ebtisam A. Elhihi, Faisal A. Alasmari, Omar K. Abdel Rahman, Fahad T. Almoallad, Reem A. Alsalhi, Shuruq F. Alosaimi, Faisal M. Alhazmi, Marwan S. Hawsawei, Ziyad A. Alasmari

**Affiliations:** ^1^ Nursing Research and Evidence-Based Practice Department King Abdullah Medical City, Makkah, Saudi Arabia; ^2^ Cardiac Care Unit King Abdullah Medical City, Makkah, Saudi Arabia; ^3^ Cath Lab King Abdullah Medical City, Makkah, Saudi Arabia; ^4^ Nursing Administration King Abdullah Medical City, Makkah, Saudi Arabia; ^5^ Day Care Department King Abdullah Medical City, Makkah, Saudi Arabia

## Abstract

**Background:**

The acute myocardial infarction mortality risk rises by 8% per year for every 30-minute delay in early coronary intervention following the onset of symptoms. Thus, it is important to reduce the door-to-balloon time as much as possible, especially in hospitals where early coronary intervention is carried out within 90 minutes.

**Aim:**

The purpose of this study was to determine the impact of King Abdullah Medical City's strategies on balloon time for patients with ST elevation myocardial infraction.

**Methods:**

Prospective observational research was conducted in King Abdullah Medical City. This study included 67 patients who had a primary percutaneous coronary intervention. Data were collected in Hajj 2023 through direct observation using a checklist that included two parts: (I) patients' demographic characteristics and relevant time intervals. The data were analyzed using descriptive statistics (frequency and percentage; median and interquartile range) and inferential statistics (Mann–Whitney *U* test, Kruskal–Wallis H test, Spearman correlation coefficient test).

**Results:**

It was noted that the median overall door-to-balloon time was 68 minutes for direct admission patients and 100 minutes (median) for interhospital transferred patients, with a statistically significant *P* value of 0.001. DTBT had no significant correlation with either the length of stay or hospital mortality rates (*P* > 0.05).

**Conclusions:**

King Abdullah Medical City accomplished an international benchmark in door-to-balloon time for ST elevation myocardial infraction patients visiting the hospital for percutaneous coronary intervention during the hajj season. Healthcare organizations can take proactive steps to optimize the management of STEMI cases. This includes establishing efficient communication channels, standardizing protocols, and facilitating seamless transitions between healthcare facilities.

## 1. Introduction

Primary percutaneous coronary intervention (PCI) is the gold standard treatment for acute myocardial infarction with ST-segment elevation. Regrettably, many patients diagnosed with acute myocardial infarction (AMI) are admitted to institutions that lack the resources to deliver prompt and appropriate acute care, including early revascularization procedures. It is mentioned that these patients be transferred to facilities with the capability to perform percutaneous coronary intervention for subsequent therapy. The American Heart Association has popularized the term door-to-balloon (DTBT) to highlight the importance of prompt PCI management. The period between a patient's arrival at a hospital and the time their balloon is inflated is recognized as door-to-balloon time. According to the guidelines, the objective DTBT is 90 minutes or less. First medical contact to device time of 120 minutes is recommended by both European and American ST Elevation Myocardial Infraction (STEMI) standards for the transfer STEMI population [1, 2].

The measurement of DTBT has emerged as a crucial metric in PCI in recent years and has been included in national guidelines as a fundamental performance indicator. Nevertheless, advancements in DTBT have not been accompanied by proportional declines in fatality rates [3]. As a surrogate for total ischemic time, some scholars have proposed symptom-to-balloon time (STB) as a more suitable metric for PCI performance [4]. In a previous study, it was demonstrated that an STB time exceeding 160 minutes is linked to a higher incidence of left ventricular dysfunction six weeks after primary percutaneous coronary intervention. Previous studies have indicated that prolonged ischemia is linked to elevated levels of oxidative stress, greater size of infarction, and heightened likelihood of unfavorable outcomes such as mortality [5].

Upon arrival at the emergency department, several strategies can facilitate the evaluation and management of STEMI situations. These include early ECG, rapid ECG interpretation, early actuation of catheterization lab, a swift activation response, and rapid reperfusion [6, 7]. During the hajj season, many pilgrims are exposed to acute coronary syndrome and transfer to King Abdullah Medical City (KAMC) for therapeutic interventions like percutaneous coronary intervention. Due to the high rate of admission, new strategies are implemented to provide high-quality care to patients with AMI as a new pathway of the patient admission, critical bed management group and chest pain unit during hajj. So, the aim of this study is to determine the impact of King Abdullah Medical City's strategies on door-to-balloon time in patients with STEMI.

## 2. Methods

### 2.1. Study Design

Prospective observational research using checklist for direct observation which included two parts, Part one: it included patients' demographic characteristics, Part two: relevant time intervals.

### 2.2. Study, Setting, and Participants

This study was conducted in King Abdullah Medical City which is one of Saudi Arabia's largest medical cities with 390 active beds capacity in Makkah. All patients undergoing PCI during hajj from 23/6/2023 to 7/7/2023 either direct admission or interhospital transfer were included. Patients who died before starting the procedure and patients whose transfer time surpassed an acceptable duration prevent them from timely reperfusion intervention were excluded. This study included a convenience sample of 67 patients. All patients provided oral consent.

### 2.3. Data Collection

After getting official permission from the KAMC, Holy Makkah IRB with the approval number 23-1092, the data were collected by the researchers through direct observation using checklist. Upon the patient's arrival, verbal agreement was sought from each patient for data collection and subsequent follow-up.

#### 2.3.1. King Abdullah Medical City Hajj' Strategies

1. New patient admission pathway implemented. The Cath lab crew and medical coordinator were notified when the physician accepted the patient for PCI by hotline. The medical coordinator informed the admission office, health information management, and bed management coordinators. The admission office accessed patient files and the bed management coordinator booked a bed. The Cath lab team prepared the theater and assigned medical staff who was responsible for the patient. PCI was ready when the patient arrived at the hospital.
2. WhatsApp group named Critical bed management group for administrative purposes aimed to check availability of beds to book beds for patients before arrival to the hospital.
3. A new crucial section, the chest pain unit, opens during Hajj season. All patients entered chest pain unit directly. This unit had three cubic with 10 beds, 3 critically ill patients' beds with mechanical ventilators, backup intubation, and 2 crash cards. Three cardiologists, two echo technicians, one respiratory therapist, one patient care technician, and two porters were also assigned. The chest pain unit had a charge nurse and three cubic nurse groups. One of the three nurses in each group is the team leader and a senior critical care professional. All patients should have fast screen echo at chest pain unit to roll out mechanical issues.

### 2.4. Instrument

The checklist included two parts: Part one covered patients' demographics such gender, age, marital status, education, diagnosis, and comorbidities. Part 2: relevant times. This study examined the time from the initial door entry to the initiation of the electrocardiogram (ECG) procedure, the time between the ECG and the final diagnosis, the time from diagnosis to the second door entry, the time from the second door entry to the confirmation of the ECG results, the time between the ECG and the confirmed diagnosis, and the time from acute myocardial infarction confirmed diagnosis to the commencement of the catheterization laboratory (Cath lab) procedure, and the duration from the Cath lab procedure to the initiation of balloon angioplasty.

The difference in time between the time of ballooning and the time of patient arrival at the first hospital was defined as the door-to-balloon time. Length of stay was calculated from the day of admission today of discharge. In hospital mortality was defined as the rate of death from any cause.

In the context of patients who present directly to KAMC, a facility equipped for Primary Percutaneous Coronary Intervention (PPCI), irrespective of whether their arrival is through self-presentation or via Emergency Medical Services, the parameter of DTBT was defined as the temporal interval from the moment of the patient's arrival at KAMC to balloon inflation time.

DTBT was defined as arrival time at the noncapable healthcare facility to balloon inflating time for patients who transferred from another facility. Medical professionals who transferred patients to KAMC provided the arrival time for PCI-incapable healthcare facilities and the first EEG time from ECG paper. The classification of DTBT was based on the following time intervals: first door to first ECG, AMI diagnosis to second door, ECG to AMI confirmed diagnosis, Cath lab arrival to balloon inflation, and AMI confirmed diagnosis to second door (PCI-capable hospital door). D1-D2 time was also calculated. Electronic health records provided hospital stay and death data. All data were tabulated in Excel.

### 2.5. Data Analysis

Data were analyzed through using Statistical Package for the Social Sciences (SPSS), version 26. The normality distribution was assessed using Kolmogorov–Smirnov test and the normality assumption was rejected (*P* < 0.05). Therefore, categorical variables were presented as frequency and percentages and continuous variables were presented as medians with interquartile ranges (IQR). The Mann–Whitney *U* test was used to assess differences between two independent groups. Whereas Kruskal–Wallis H test was used to assess differences between more than two independent groups. The Spearman correlation coefficient test was employed to assess the strength and direction of associations between nonparametric variables. Statistical significance was set at *P* < 0.05.

## 3. Results

Table 1 shows demographic characteristics of the patients, it was observed that majority of patients were male, married, and aged more than 50 years old (91%, 98.5%, and 83.6%, respectively). 14.9% of patients were Saudi and 13.4 were Indian. Regarding body mass index, it was found that 88.1% of patients had normal body mass index. 76.1% of patients transfer from another healthcare facility and 25.4% of them transfer from Alnoor hospital.

Table 2 presents patients' health-relevant data, regarding past medical history it was noted that more than half of patients (52.2%) had hypertension, 47.8% of patients had diabetes and 14.9% of patients had history of angiography and PCI. It was noted that more than one third of patients (46.3%) had right coronary artery occlusion with inferior MI and 31.3% of patients had normal left ventricle ejection friction. Moreover, 6% of patients had Cardiopulmonary resuscitation, 4.5% of them died.

Table 3 shows the parameters of DTBT for studied patients. It was noted that the median overall door-to-balloon time was 68 minutes for direct admission patients and 100 minutes for interhospital transferred patients with statistically significant, *P*=0.001. The median time from diagnosis of AMI to Cath lab for direct admitted patients was 36.50 minutes and 60 minutes for transferred' patients with statistically significant, *P*=0.001.

**Figure 1:**
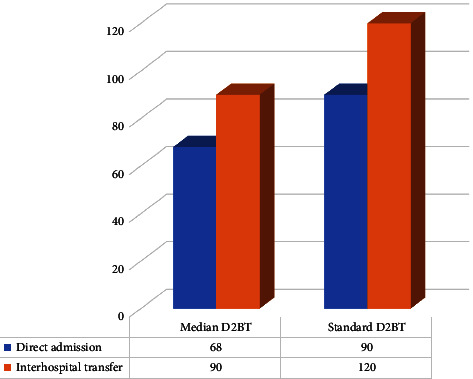
Comparison of DTBT with the standard time between direct admission and interhospital transfer.

Figure 1 illustrates the comparison of DTBT with the standard time between direct admission and interhospital transfer. The median of DTBT for direct admitted' patients was found to be less than the standard time of DTBT for PCI-capable hospital (68 m to 90 m, respectively). For interhospital transfer patients median DTBT was 100 m compared with the 120 m for the standard time.

Table 4 presents the time spent from patients' diagnosis to balloon for interhospital transfer patients. It was noted that the median time from AMI diagnosis to second door (door of capable hospital) was 47 minutes and from arrival to capable hospital to balloon inflation was 28 minutes. The median time from D1 to D2 was 65 minutes and median time from D2 to balloon (patient arrival to KAMC to balloon) for interhospital transferred patients was 28 minutes which is within the recommended guidelines for patients transfer time.

Table 5 reveals that DTBT had no significant correlation with either the length of stay or hospital mortality rates.

Table 6 presents that no statistically significant variations were found between demographic data of the studied patients and door-to-balloon time except name of hospitals (*P* < 0.05).

## 4. Discussion

This study aimed to determine the impact of King Abdullah Medical City's strategies on balloon time for patients with ST elevation myocardial infraction. All relevant clinical guidelines agree that PPCI is the most effective early therapy for patients experiencing a STEMI and that rapid PCI is the most effective early therapy for patients experiencing a high-risk or very high-risk non-ST-segment elevation myocardial infarction (NSTEMI). Patients presenting with STEMI or highly high-risk NSTEMI should be moved to a PCI-capable institution within 120 minutes, as recommended by current recommendations [2, 8, 9].

Our study found that the median DTBT for STEMI patients either direct admission or transfer was within the guidelines recommended time, this may be due to efficient coordination and communication between hospitals, sufficient resources, and staffing levels to handle interhospital transfers efficiently, dedicated transfer protocols implementation using aircraft and emergency medical services and availability of cardiac catheterization labs at KAMC. The findings of this study align with a previous investigation conducted in Saudi Arabia by Butt et al. at a tertiary care institution in Riyadh. This study's purpose was to outline various interventions, collect data for the designated study period, address the challenges associated with ensuring round-the-clock patient access to PCI, and evaluate quality indicators. This study concluded that for individuals presenting with STEMI in the emergency department, PCI is the preferred therapeutic approach. Furthermore, the King Faisal Specialist Hospital and Research Centre in Riyadh has successfully attained and sustained an international benchmark of DTBT within 90 minutes through effective multidisciplinary collaboration [10].

Interhospital transfer patients were observed to have a shorter admission to balloon time than direct admitted patients. This discrepancy may be attributed to the initial assessment conducted at the referring hospital, which aids in discerning whether the patient was diagnosed with STEMI prior to their subsequent transfer to PCI-capable medical center, patients were admitted directly to chest pain unit, available medical staff waiting patient' arrival, patient's medical file was prepared, and Cath lab teams are already at the hospital, waiting for the patients instead of needing to come in from their homes. This result is in line with Hu et al. and Kawecki et al. who reported that the patients who were transported had a shorter DTBT than those who arrived directly at hospitals with PCI capabilities [11, 12].

The present study's findings reveal that the median DTBT for direct admitted' patients at KAMC were found to be less than the standard time and PCI was performed within 28 minutes from interhospital transfer patients' arrival to hospital door. This could be due KAMC strategies that focus on implementation of streamlined protocols, effective communication between healthcare providers, effective utilization of technology, optimal resource allocation, optimized patient flow, and prioritization of high-risk cases which result in a shorter DTBT. This result is supported by Ravi et al., Nathan et al., and Dhungel et al., who reported that the DTBT was well within the current American College of Cardiology and American Heart Association guideline recommendation [13, 14]. Bypassing unnecessary admission to the chest pain unit and directly transferring patients from the ambulance to the catheterization lab can be an effective approach to expedite reperfusion therapy.

The present study demonstrates that there is no statistically significant relationship between total door-to-balloon time and length of stay or total door-to-balloon time and in-hospital mortality. The observed phenomenon may be attributed to the constrained sample size, which has resulted in restricted statistical power to identify major disparities and developments in medical practices during the hajj season. This result is supported by Fan et al. [15] who reveal that there are no statistically significant differences between in-hospital mortality rate and D2B time. In contrast, Chew et al., Park et al., and Foo et al. found that delay in primary PCI could lead to increase in-hospital mortality [16, 17]. Moreover, Li et al. reported that patients with ST-elevation myocardial infarction had a strong association between hospital costs and length of stay [18, 19].

The findings of this study demonstrated that there were statistically significant differences between access to hospitals and the DTBT. This may be due to the proximity and accessibility of hospitals that play a crucial role in DTBT. Patients admitted directly to the hospital have shorter travel times that reduce the overall DTBT. On the other hand, patients transferring from remote areas or facing transportation challenges may experience delays in reaching the hospital, leading to longer door-to-balloon times.

The findings of the study have significant implications for clinical practice, highlighting the crucial role of effective coordination, streamlined procedures, and prompt access to institutions equipped for PCI in lowering DTBT and enhancing outcomes for patients with STEMI. Further investigation is needed to examine the factors that affect DTBT and patient outcomes to improve reperfusion techniques and enhance the quality of treatment for individuals with acute myocardial infarction. Furthermore, studies investigating the potential of new technology like artificial intelligence and telemedicine to enhance DTBT procedures may provide fresh ideas for improving the effectiveness and efficiency of treatment delivery.

### 4.1. Limitations

Despite the study limitations firstly as a single-center observational study with a limited sample size and heterogeneity within the study population due to a significant influx of pilgrims from diverse geographic regions and backgrounds during hajj season, this study accurately reflects the clinical realities of PPCI procedures throughout the hajj season. The results of KAMC strategies are applicable to other regions. Secondly, the time of the patients' transfer from a noncapable hospital was not available (door out), so door in and door out time could not be evaluated. Thirdly, there may have been an inherent selection bias in the enrollment process, as the study exclusively encompassed patients possessing comprehensive information pertaining to DTBT, their arrival at a PCI-capable facility, and their subsequent participation in PCI.

## 5. Conclusions

The KAMC was able to accomplish a DTBT that set an international benchmark for STEMI patients who presented to the hospital for PCI during the hajj season, primarily through the implementation of strategic approaches to decrease DTBT. By implementing KAMC strategies, the processes of diagnosis, decision-making, and patient transfers will be executed in a synchronized and expeditious manner, resulting in improved patient care and less suffering. Further research into the effects of symptom onset, initial contact with a medical provider or balloon time on clinical outcome is also required.

## Figures and Tables

**Table 1:** Demographic characteristics of the studied patients.

Characteristics	*N*	%
Age		
(i) 30–39	2	3.0
(ii) 40–49	9	13.4
(iii) >50	56	83.6
Gender		
(i) Male	61	91.0
(ii) Female	6	9.0
Nationality		
(i) Saudi	10	14.9
(ii) Pakistani	8	11.9
(iii) Indian	9	13.4
(iv) Turkish	8	11.9
(v) Indonesian	5	7.5
(vi) Egyptian	6	9.0
(vii) Others	21	31.3
Marital status		
(i) Married	66	98.5
(ii) Single	1	1.5
Body mass index		
(i) Normal	59	88.1
(ii) Overweight	3	4.5
(iii) Obese	5	7.5
Access to hospital		
Direct admission	16	23.9
Transfer	51	76.1
Name of referral hospital		
(i) Alnoor hospital	13	25.49
(ii) Hajj mission	12	23.52
(iii) King Abdulaziz hospital	5	9.80
(iv) Mina hospital	5	9.80
(v) Arafat hospital	3	5.88
(vi) King Faisal hospital	5	9.80
(vii) Others	8	15.68

**Table 2:** Health profile of the studied patients.

Past history		
(i) Hypertension	35	52.2
(ii) Diabetes	32	47.8
(iii) Smoking	12	17.9
(iv) Dyslipidemia	8	11.9
(v) History of angiography	10	14.9
(vi) History of PCI	10	14.9
Procedural characteristics		
(1) Type of myocardial infraction		
(i) Anterior MI	28	41.8
(ii) Inferior MI	31	46.3
(iii) Posterior MI	6	9.0
(iv) Lateral MI	2	3.0
(2) Patients' blocked arteries		
(i) Right coronary artery	31	46.3
(ii) Left main coronary artery	1	1.5
(iii) Left anterior descending coronary artery	30	44.8
(iv) Left circumflex coronary artery	5	7.5
Left ventricle ejection friction		
(i) Normal	21	31.3
(ii) Mild dysfunction	20	29.9
(iii) Moderate dysfunction	18	26.9
(iv) Severe dysfunction	8	11.9
In hospital clinical outcomes		
(i) Cardiogenic shock	3	4.5
(ii) Death	3	4.5
(iii) Cardiopulmonary resuscitation	4	6.0

**Table 3:** Comparison door-to-balloon time between direct admission and interhospital transfer for the studied patients.

	Direct admission *N* (16)		Interhospital transfer *N* (51)		*Z*
	Median	IQR	Median	IQR	
(1) Door-to-ECG time (m)	10.0	4.0	10.0	0.00	2.36^∗∗^
(2) ECG to AMI diagnosis time (m)	4.5	7.0	6.0	15.0	1.36
(3) Diagnosis of AMI to cath lab time (m)	36.50	45.50	60.0	30.0	3.42^∗∗^
(4) Cath lab to balloon time (m)	18.50	16.50	15.0	10.0	1.58
(5) Door-to-balloon time (m)	68.0	33.0	100.0	39.0	3.44^∗∗^

*Z*: Mann–Whitney *U* test/^∗∗^significant at *P* < 0.05.

**Table 4:** The time spent from patients' diagnosis to balloon for interhospital transfer patients.

	Median	IQR
(1) AMI diagnosis time to D2	47.00	35.00
(2) D1 to D2	65.00	40.00
(3) D2 to balloon	28.00	13.00

**Table 5:** Relationship between door-to-balloon time and length of stay and hospital mortality.

Variables	Door-to-balloon time	
	*r*	*P*
Length of stay	0.09	0.42
Hospital mortality	0.10	0.39

**Table 6:** Relationship between the demographic data and door-to-balloon time.

Characteristics	Door-to-balloon time		Test of significance
	Median	IQR	Chi-square/*Z*
Age years			
(i) 30–39	144.00	51.00	0.55
(ii) 40–49	95.00	43.00	
(iii) >50	93.50	45.25	
Gender			
(i) Male	95.00	42.00	0.19
(ii) Female	92.00	72.25	
Nationality			
(i) Saudi	79.00	24.50	4.14
(ii) Pakistani	88.50	23.75	
(iii) Indian	81.00	44.00	
(iv) Turkish	84.00	94.00	
(v) Indonesian	106.00	163.00	
(vi) Egyptian	99.50	69.50	
(vii) Others	104.00	47.50	
Body mass index			
(i) Normal	92.00	36.00	1.95
(ii) Overweight	110.00	75.00	
(iii) Obese	120.00	122.50	
Type of myocardial infraction			
(i) Anterior MI	91.00	44.25	0.13
(ii) Inferior MI	99.00	41.00	
(iii) Posterior MI	92.50	86.00	
Patients' blocked arteries			
(i) Right coronary artery	100.00	32.00	
(ii) Left anterior descending coronary artery	82.00	46.25	4.73
(iii) Left circumflex coronary artery	84.00	34.00	
Name of hospitals			
(i) Alnoor hospital	110.00	103.50	16.08^∗^
(ii) King Abdullah medical city	68.00	33.00	
(iii) Hajj mission	83.50	92.25	
(iv) King Abdulaziz hospital	104.00	19.00	
(v) Mina hospital	105.00	86.50	
(vi) Arafat hospital	100.00	56.00	
(vii) King Faisal hospital	101.00	40.50	
(viii) Others	85.00	24.50	

Chi-square: Kruskal–Wallis *H* test/*Z*: Mann–Whitney *U* test/^∗^*P* < 0.05.

## Data Availability

The data that were used and analyzed to support the findings of this study are available from the corresponding author on request.
